# Implications of S-glutathionylation of sarcomere proteins in cardiac disorders, therapies, and diagnosis

**DOI:** 10.3389/fcvm.2022.1060716

**Published:** 2023-01-24

**Authors:** Paola C. Rosas, R. John Solaro

**Affiliations:** ^1^Department of Pharmacy Practice, College of Pharmacy, Chicago, IL, United States; ^2^Department of Physiology and Biophysics, College of Medicine, University of Illinois at Chicago, Chicago, IL, United States

**Keywords:** myosin binding protein C, titin, cardiac troponin I, protein phosphorylation, cross-bridge kinetics, length dependent activation

## Abstract

The discovery that cardiac sarcomere proteins are substrates for S-glutathionylation and that this post-translational modification correlates strongly with diastolic dysfunction led to new concepts regarding how levels of oxidative stress affect the heartbeat. Major sarcomere proteins for which there is evidence of S-glutathionylation include cardiac myosin binding protein C (cMyBP-C), actin, cardiac troponin I (cTnI) and titin. Our hypothesis is that these S-glutathionylated proteins are significant factors in acquired and familial disorders of the heart; and, when released into the serum, provide novel biomarkers. We consider the molecular mechanisms for these effects in the context of recent revelations of how these proteins control cardiac dynamics in close collaboration with Ca^2+^ fluxes. These revelations were made using powerful approaches and technologies that were focused on thin filaments, thick filaments, and titin filaments. Here we integrate their regulatory processes in the sarcomere as modulated mainly by neuro-humoral control of phosphorylation inasmuch evidence indicates that S-glutathionylation and protein phosphorylation, promoting increased dynamics and modifying the Frank-Starling relation, may be mutually exclusive. Earlier studies demonstrated that in addition to cTnI as a well-established biomarker for cardiac disorders, serum levels of cMyBP-C are also a biomarker for cardiac disorders. We describe recent studies approaching the question of whether serum levels of S-glutathionylated-cMyBP-C could be employed as an important clinical tool in patient stratification, early diagnosis in at risk patients before HFpEF, determination of progression, effectiveness of therapeutic approaches, and as a guide in developing future therapies.

## 1. Introduction: S-glutathionylation as a potential modifier of sarcomere protein function

In view of evidence discussed below that cardiac myosin binding protein C (cMyBP-C), actin, cardiac troponin I (cTnI) and titin are modified by S-glutathionylation, we focus here on these sarcomere proteins in physiological control of cardiac function by this oxidative related and potentially maladaptive post-translational modification. A prevalence of reduced glutathione (GSH) is critical to maintain the reducing environment in cardiac myocytes. With a loss of redox balance and exposure of proteins to reactive oxygen species (ROS), there is a promotion of a reversible oxidation of targeted Cys residues that may be adaptive or maladaptive (1). Redox homeostasis and modifications of the cardiac myocyte depend strongly on a balance between reduced (GSH) and oxidized glutathione (GSSG). Reaction of GSSG with proteins, which is reversible, may be beneficial by protecting Cys residues from irreversible modification such as carbonylation (1). However, with persistent and severe oxidative stress the balance of GSH/GSSG tilts unfavorably leading to pathological consequences in a variety of mechanisms as reviewed by Pastore and Piemonte (1). Although early studies emphasized the significance of S-glutathionylation in cell signaling involving myofilament proteins, the functional significance of this post-translational modification in cardiac sarcomeres remained unclear (2, 3). Our discovery reported in 2012 of a role of S-glutathionylation of cMyBP-C in control of sarcomere response to Ca^2+^ and cross-bridge kinetics provided a new understanding of the effects oxidative stress on cardiac function especially diastolic function (4). Our findings were confirmed and extended by other laboratories. However, more work needs to be carried out to understand the role of S-glutathionylation more completely in early and late stages of cardiac disorders, in therapies, and in their usefulness as serum biomarkers in diagnosis and stratification of patients.

Advances in the understanding of molecular control processes in sarcomere function demand a new look at the integrated interactions among the proteins modified by S-glutathionylation in mechanisms of control of the heartbeat and beat-to-beat regulation. With the development of better high-resolution imaging techniques such as cryo-electron microscopy (cryo-EM) (5) together with atomistic molecular modeling (6), molecular-dynamics (MD) simulations (7, 8), and interrogating protein-protein interactions with fluorescent probes (9, 10), there have been significant investigations contributing to a new and dynamic evolution in understanding the structure and function of all three of the filaments of the cardiac sarcomere. Detailed accounts of these advances are in recent publications to which we refer in the present paper in the context of our focus on oxidative modifications of cMyBP-C, actin, cTnI, and titin by S-glutathionylation.

## 2. Transitions in thin, thick, and titin filament states in the heartbeat and beat to beat regulation

### 2.1. The diastolic states and transitions to systole

**FIGURE 1:**
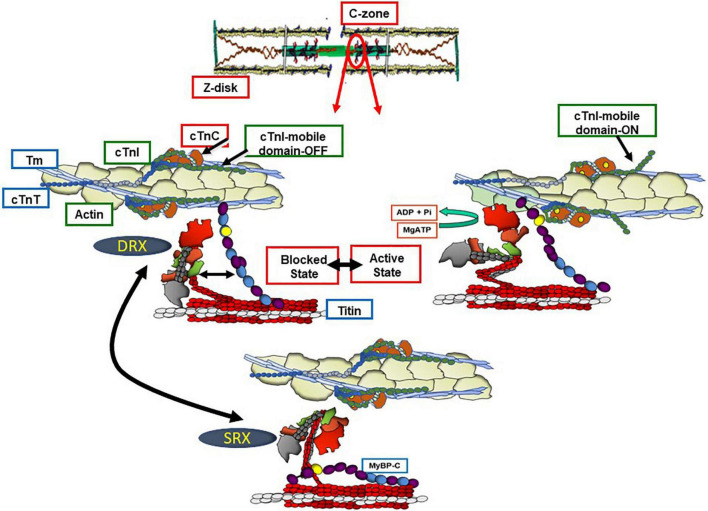
Functional units of C-zone sarcomeres in relaxed and active states. Shown are functional units of cardiac sarcomeres in the C-zone containing c(MyBP-C). The **bottom panel** shows a sarcomere the Ca-regulatory site on cTnC not occupied and with myosin in a super relaxed state (SRX) in which the heads are sequestered and not participating in the heartbeat. In the **left middle panel**, the cross-bridges are in a disordered relaxed state (DRX) poised to interact with the thin filament but blocked by the actions of the troponin complex proteins with an inhibitory peptide (Ip) and mobile domain of troponin I (cTnI) locking tropomyosin (Tm) a position sterically blocking the actin-cross-bridge interaction. This blocking position of Tm relies on an interaction of troponin T (cTnT) with the overlap region of Tm. In this state there is an interaction of the N-terminal domain of myosin binding protein C (cMyBP-C) with actin-Tm moving Tm away from it’s steric blocking position. As indicated, there are also interactions between the middle regions of cMyBP-C and myosin producing a drag on shortening capability. The **right middle panel** shows that with Ca-binding to the N-terminal domain of troponin C (cTnC) the thin filament is released from inhibition permitting activation of cross-bridge interactions with actin promoting force and shortening of sarcomere. The kinetics of the actin-cross-bridge reaction depend on interactions with the N-terminus of cMyBP-C (see text and Figure 2 for discussion).

Figure 1 illustrates integrated function of proteins in the sarcomere filaments in establishing the diastolic and systolic states. We first discuss a current perception of the transition between these states in a basal level of contractility and heart rate, in which there exists a contraction and relaxation reserve. Shown are functional units in a sarcomere consisting of thin filaments with a 7:1:1 ratio of actins: tropomyosin and troponin (Tn) complex (cTnI), the inhibitory unit; cTnC, the Ca-binding unit, and cTnT, named for its binding to tropomyosin (Tm). Sarcomeres illustrated in Figure 1 are in the C-zone, where cMyBP-C localizes (11). Titin, which stretches from the Z-disk to the M-band and establishes passive tension in diastole is shown extending from the sarcomeres in Figure 1 and discussed in detail below. In each functional unit, Figure 1 shows the head, hinge, and extended tail of two headed myosin (cross-bridges) with associated light chain proteins. The reaction of myosin cross-bridges with actin generates tension, shortening, and thus the power to eject blood. In relaxed sarcomeres thin filaments are in a “B” or myosin blocked state. cMyBP-C is in the central so-called C-zone of the sarcomere, and as discussed below interacts both with the thin, thick, and titin filaments. At end diastole, sarcomere length is relatively long, the stretch of titin elastic domains induces resting tension and diastolic pressure (12). In the B-state, there is a dominant obstruction of Tm movement away from its blocking configuration resulting in inhibition of the actin-cross-bridge reaction (13). This inhibition depends on weak interactions with the cTnI-switch peptide (SwP) with cTnC and strong interactions of the cTnI inhibitory peptide (Ip) and C-terminal mobile domain extending from the cTn core domain and holding Tm in a blocking position. There are also interactions of cTnI-cTnC with a cTnT peptide known as the IT arm. Relaxation also depends on a peptide of cTnT located at the overlap of contiguous Tms. As emphasized by Tobacman (14), recent studies of the thin filament structure revealed that each of the cTns on opposite faces of the thin filament interact with both Tm strands. The Tn complexes on opposite sides the thin filament are not in perfect register. It is now recognized that processes at the level of the thick filament proper also play a role in the relaxed states. Evidence has shown that cardiac myosin can exist in either a sequestered super-relaxed state (SRX) with very low ATPase activity with the heads folded back toward the thick filament, or in a disordered relaxed state (DRX) with heads poised to interact with thin filaments in the transition to the active state of the actin-myosin cross-bridge reaction (15, 16). Physiological and pathological mechanisms modulate the population of these states in thick filament related control processes (15).

With its release into the sarcoplasm, Ca^2+^ binds to the N-lobe of cTnC promoting a tight binding of cTnI SwP and releasing the tether of the mobile domain to actin; the N-lobe of cTnC now binds to actin-Tm in association with a pivot of the core domain of cTn (5, 7, 14). Current theories suggest that these actions of the cTn complex of proteins induce a Ca-activated or “C” state but do not fully release the thin filament from inhibition. A requirement for interactions of the cross-bridge with actin-Tm has been proposed to move Tm fully from its obliteration of myosin binding inducing a myosin activated or “M” state (13). Although not indicated in Figure 1, activation of a functional unit may induce cooperative spread of activation along the thin filament to other functional units (17).

**FIGURE 2:**
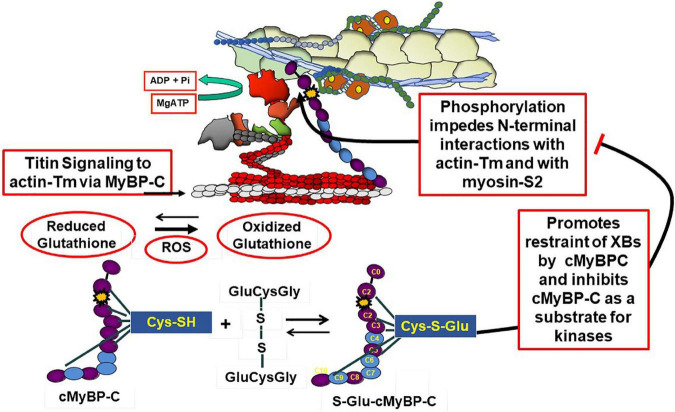
Modulation of cMyBP-C by phosphorylation and oxidative stress. The **top panel** shows an active sarcomere emphasizing cMyBP-C interactions at the N-terminal domains with the thin filament and myosin and C-terminal domains interacting with tails of myosin and titin. These interactions are inhibited with site specific phosphorylations (yellow star) of an N-terminal domain (yellow star) and modulated by oxidative stress demonstrated by the action reactive oxygen species (ROS) to increase the ration of reduced glutathione to oxidized glutathione. As shown the oxidized glutathione reacts with available Cys residues generating S-glutathionylated (S-Glu) cMyBP-C, which, as discussed in the text, may block the effects of phosphorylation to increase cross-bridge kinetics. Cys residues identified in various studies (41, 42, 57) are shown to exist in N-terminal and C-terminal regions as well as domains in the **middle panel**.

Complex interactions among titin, cMyBP-C and the thin filament in control of contraction and relaxation dynamics and in the Frank-Starling relation are in a state of evolution in our understanding and require discussion. A comprehensive summary of mechanisms of cMyBP-C structure and function has been recently published by Suay-Corredera and Alegre-Cebollada (18). Interactions of cMyBP-C with thin, thick, and titin filaments provide an array of mechanisms to maintain homeostasis over a range of physiological states. These mechanisms are associated with domains in N-terminal, central, and C-terminal domains. As emphasized by the studies by Mun et al. (19), these regions of cMyBP-C control sarcomere activity by multiple and independent mechanisms. Moreover, these interactions comprise sites of vulnerability associated with familial cardiac disorders (20). Figures 1, 2 depict a current perspective the disposition of cMyBP-C in regions of a sarcomere with functional units in various states of activity. Ten regions consisting of fibronectin III (FN) and immunoglobulin (Ig) domains are indicated. Unique aspects of cMyBP-C are indicated at the N-terminal domain with phosphorylation sites and in an Ig domain with a loop present in the middle of the cardiac isoform (Figure 2). Cys residues identified to be modified by S-glutathionylation are also indicated and discussed below. It is accepted that that cMyBP-C extends from C-terminal anchoring sites on titin and light meromyosin (LMM) across the inter-filament space to the thin filament with N-terminal sites binding alternatively with the head-neck region of myosin and actin-Tm (21–23). At low Ca^2+^ evidence indicates that the flexible N-terminus of cMyBP-C scans the thin filament seeking binding sites (24, 25). Binding of the N-terminus of some cMyBP-C molecules to actin-Tm moves Tm away from full inhibition, thereby activating the thin filament independently of the Ca-cTnC interaction (26, 27). Regions of cMyBP-C in the central middle domains have multiple functional roles acting in concert with N- and C-terminal domains. Importantly, compared to other regions, middle regions C2C4 and C5C7 have the highest affinity for tail regions of myosin S2 with both regions binding strongly with myosin light chains attached. C5C7 binds strongly to this S2 regions even without the myosin light chains present (28). There is evidence for multiple mechanisms modified by these interactions that have functional effects on sarcomere activity. These mechanisms, which modulate power and energy consumption, include the generation of an internal load inducing a drag on sarcomere shortening, an inhibition of actomyosin ATPase activity, and an induction of the SRX state (29, 30). There is also a proposal that the interaction between central domains of cMyBP-C with myosin steers cross-bridges to and from thin filaments thereby controlling the rate of recruitment and exit from force generating state (31). C-terminal interactions modulate thick filament packing and specific interactions of 3 C-terminal domains with specific super-repeats on titin establish localization of cMyBPC in its striped configuration in the C-zone of the sarcomere (32). As discussed below, there is evidence that these interactions are modulated by mechanical stress and by phosphorylation. There are also interactions of cMyBP-C domains with the regulatory light chain of myosin in a phosphorylation dependent manner (33). In summary, it is apparent that the population of cMyBP-C molecules in a sarcomere consists of some with N-terminal flexible regions interacting with the thin filament and others interacting with various regions of thick filament proteins slowing cross-bridge kinetics and some inducing an SRX state (34). Adding to the complexity of the disposition of proteins in relaxed and active sarcomeres is evidence, that these interactions are biased toward interactions with actin-Tm in the low Ca^2+^ inhibited state of the thin filament and more toward interactions with myosin heads at activating levels of Ca^2+^. As discussed below these interactions are also modified by phosphorylation and by titin induced strain with stretch.

**FIGURE 3:**
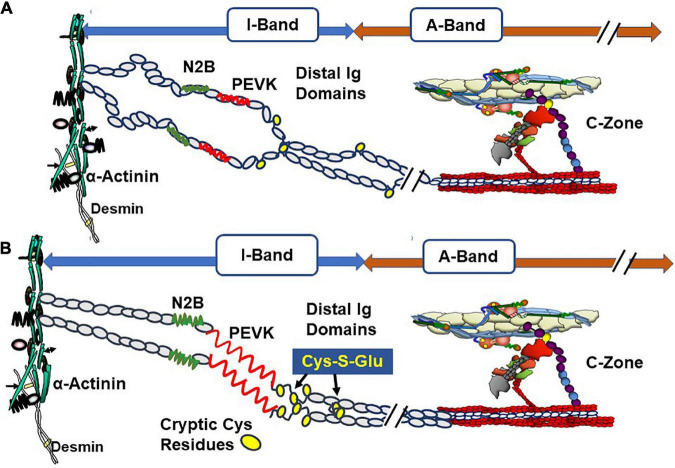
Oxidative stress and length dependent stabilization of an unfolded state of titin distal Ig domains induced by exposure and S-glutathionylation of cryptic cysteine residues. **(A)** Illustration of stretch of titin in diastolic conditions with oxidant stress at a relatively short sarcomere length showing few exposed cysteines (yellow circles). **(B)** Illustration of stretch of titin with oxidant stress to a relatively long sarcomere length showing exposure of buried cysteine residues and S-glutathionylation. This figure is based on evidence reported by Loescher et al. (12) demonstrating that with mechanical and oxidant stress, stabilization of the state of distensibility of titin occurs with aggregation of distal Ig domains of parallel titin strands. Variations in this mechanism produce a range of values of passive tension and can affect length dependent activation (LDA). Evidence indicates that stretch of titin signals interactions with the C-terminal domains of cMyBP-C that are transmitted to the cMyBP-C N-terminal domains, which promote interactions with actin-Tm and activation of the thin filament. See text for further discussion.

Titin is the major protein establishing passive tension in the diastolic state but also may be a participant in feedback signaling from the systolic state to the thick filament cross-bridges and signaling in communication with cMyBP-C and the thin filament (35–37). Regions of titin that are significant in passive tension are illustrated in Figure 3. Recent reviews provide a picture of this giant protein and its modulation by stretch, phosphorylation, and oxidant stress (12, 38).

As discussed below, complex interactions among titin, cMyBP-C, and the thin filament, which occur in control of contraction and relaxation dynamics and in the Frank-Starling relation, are in a state of evolution in our understanding. This discussion includes descriptions of accessible Cys residues expressed in each of these proteins that are targeted by S-glutathionylation. Addition of the negatively charged GSH tripeptide modifies function alters signaling *via* protein phosphorylation.

### 2.2. Beat-to-beat regulation and the Frank-Starling relation

An emerging concept under discussion in the literature is related to the signaling pathway from titin to cMyBP-C and the thin filament in their roles in length dependent activation (LDA), the basis of the Frank-Starling relation. Elegant studies by Reconditi et al. (35) employed X-ray diffraction and mechanics to reveal that a length dependent mechanism governs entry of cross-bridges into the systolic state. They elucidated the concept that a mechano-sensing mechanism rapidly responds to changes in systolic peak force and induces variations in the number of myosin heads in the off state in diastole thereby matching ejection to the hemodynamic task. In this concept of the Frank-Starling law, cross-bridges sense thick filament stress in systole, which in turn introduces variations in the number cross-bridges in the off state. Reconditi et al. (35) speculate that interactions among titin, MyBP-C, and cross-bridges may form the stress sensing mechanism. This view leaves open the question of how modifications in the thin filament affect LDA. A different view has been presented by Irving and Craig (36) in discussing the X-ray diffraction studies together with evidence from studies using fluorescent probes to monitor changes in thin and thick filaments. Ait-Mou et al. (39) employed X-ray diffraction and reported molecular rearrangements in both thin and thick filaments with changes in length. Fluorescent probe on cTnC confirmed these effects in skinned single cells. This signaling was absent in myofilaments containing a highly compliant splice variant of titin, which generated relative low passive tension. Zhang et al. (9) used polarization of probes on cTnC and MLC2 in skinned cardiac preparations and concluded that whereas length changes in peak tension mainly affect the thick filament, length changes at sub-maximal levels of activation mainly affect the thin filament. An important finding in the study of Zhang et al. was that the changes in the thin filaments occurred independently of active interactions of cross-bridges with the thin filament. In our studies (10), carried out in collaboration with Wenji Dong’s laboratory, we employed fluorescence resonance energy transfer (FRET) to probe cTnI interactions with cTnC in skinned fiber preparations containing wild-type titin or highly compliant titin in recombinant binding motif 20 (RBM20) deficient mice. Results showed that the cTnI-cTnC interaction senses sarcomere length with a dependence on the strain induced by stretch of titin. Recent studies report no LDA in skinned fiber preparations from human heart samples containing a truncating titin mutant (40). Based on all these observations, a unifying theory, depicted in Figure 3, of LDA discussed by Irving and Craig (36) is that LDA involves mechanisms involving all three sarcomere filaments. The idea is that with stretch interactions of regions of titin with cMyBP-C induce a steering of its N-terminal domain toward the thin filament, where it interacts with actin-Tm to modulate Ca-response independently of cross-bridge interactions. This mechanism may work in conjunction with strain dependent effects on thick filaments depending on the level of Ca-activation. Mamidi et al. (26) concluded from their studies that at maximum tension, LDA is dominated by feedback effects of active tension on entry of cross-bridge into force generation, whereas at submaximal levels of activation LDA is dominated by altered Ca-sensitivity. Data demonstrating that adult cardiac myofilaments regulated by slow skeletal TnI, the fetal/neonatal isoform, show blunted LDA (41) provide support for this idea. Moreover, LDA is significantly blunted in muscle preparations with ablation of cMyBP-C (42). The concept includes the idea that a diastolic mechanism governed by modifications in titin, thick filaments, MyBP-C, and troponin-tropomyosin engages ahead of and controlling the systolic state.

## 3. Protein phosphorylation as a major mechanism modifying sarcomere function

There are data indicating that S-glutathionylation and phosphorylation of sarcomere proteins may be mutually exclusive PTMs or at least need to be considered in integrative control of cardiac function in oxidative stress (43–45). Post-translational modifications of cMyBP-C especially by neural and hormonal regulation of protein phosphorylation by kinases and phosphatases modulate its interactions in complex mechanisms (46–49). Figure 2 illustrates phosphorylation dependent modifications of interactions of cMyBP-C with its neighbors in the sarcomere. Site specific phosphorylations have been identified at Ser 275, 284, and 304 in the human sequence and a hierarchy of mechanisms defined stressing a role of cMyBP-C in integration of diverse signaling cascades (49, 50). Importantly, as shown in Figure 2, phosphorylations release the inhibitory effect of cMyBP-C on cross-bridge kinetics and impeded interactions of the N-terminal domain with actin-Tm (30, 46–48).

**FIGURE 4:**
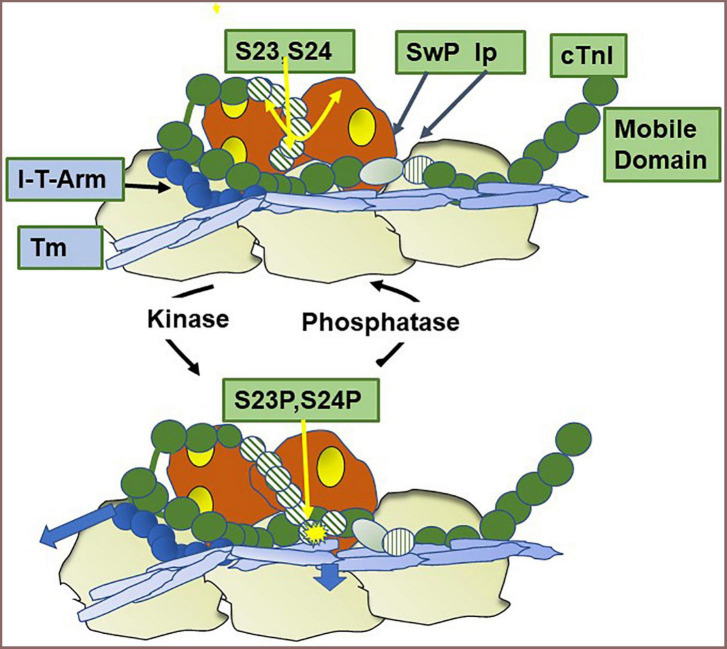
Modulation of thin filament activation by cTnI N-terminal phosphorylation. Shown is a cartoon based on recent studies (7) of a region of a Ca-activated thin filament showing the effect of phosphorylation of Ser 23 and Ser 24 in the unique N-terminal peptide of cardiac troponin I (cTnI). In the dephosphorylated state the N-terminal peptide is mobile as indicated by the double yellow arrow. Also shown are the positions of the cardiac troponin I switch peptide (Sw), inhibitory peptide (Ip), and troponin I-troponin T interaction (I-T arm) described in Figure 1. Phosphorylation induces an interaction of the N-terminal peptide with actin-tropomyosin and as shown by the arrow in the figure moves tropomyosin (Tm) to a position favoring the off state of the thin filament, thereby decreasing myofilament response to Ca^2+^ and promoting relaxation. Cys residues reported to be S-glutathionylated (42) are located in the region of the I-T arm (Cys-80) and in a helix N-terminal to the Ip (Cys 97). See text for further discussion.

cTnI is unique among isoforms in expressing a ∼30 amino acid N-terminal peptide containing two sites of phosphorylation (Ser 23, Ser 24 in the human sequence). Phosphorylation of these sites reduces myofilament Ca-sensitivity and enhances relaxation (51, 52). Figure 4 shows results of experiments revealing a new concept of thin filament regulation by the unique N-terminal peptide of cTnI (7) that has modified concepts of mechanisms of its control of sarcomere kinetics (52). Cys residues in cTnI that may participate in S-glutathionylation are indicated (44). Building on previous solution NMR structures, sequence analysis, and molecular dynamic simulations (8, 53) together with the Yamada et al. (5) cryo-EM structures of the thin filament, Pavadai et al. (7) reported the novel demonstration of interactions of a pseudo-phosphorylated N-cTnI at Ser23 and Ser24 wiith actin-Tm. They employed protein-protein docking and molecular dynamic simulations of the disposition of N-cTnI in Ca-activated thin filaments functional units. Pavadai et al. (7) concluded that together with Arg residues in the N-terminal peptide, phosphorylated Ser 23, Ser 24 act to hinder an electrostatic interaction between cTnC and Tm in the Ca-activated state. In this disposition, phosphorylated cTnI interacts with actin-Tm moving it away from its steric blocking position thus affecting relaxation kinetics without affecting maximum tension as previously reported by Cheng et al. (8). This mechanism does not include an effect of cTnI phosphorylation on SwP peptide interactions with cTnC, an effect included in early theories. These findings alter thinking regarding the role of cTnI phosphorylation in affecting the binding of the SwP to cTnC, which was not seen in the studies of Pavadai et al. (7).

As expected, phosphorylation of cardiac titin presents as a complex array of sites with variable effects on passive tension. These have been extensively reviewed and related to cardiac disorders in a review by Koser et al. (38). Important findings summarized are that PKCα and CaMKII dependent phosphorylations that occur near the PEVK domain depress passive tension, whereas PKA, cGMP, and CamKII dependent phosphorylations occur in the N2B domains and decrease passive tension. As summarized by Koser et al. (38) large changes in phosphorylation in these sites occur in HFpEF. Moreover, enhancing cGMP dependent phosphorylation of titin has been considered as a therapeutic target in HFpEF (54).

Changes in passive tension induced by titin phosphorylation in conjunction with phosphorylation of cMyBP-C and cTnI are also expected to influence the LDA signaling mechanisms discussed above. Concepts related to this signaling cascade discussed include an effect of protein phosphorylation of cMyBP-C and cTnI on the “gain” in the process of communication of the energies of activation from titin stretch to thin filament Ca-sensitivity. Konhilas et al. (55) reported an enhancement of LDA by cTnI phosphorylation and a blunting of LDA in skinned fibers regulated by slow skeletal TnI. Along these lines, Shaffer et al. demonstrated that phosphorylation of N-terminal restudies of cMyBP-C blunted LDA, a finding corroborated by studies of Mamidi et al. (56). Kumar et al. (57) investigated relative roles of cTnI and cMyBP-C phosphorylation on LDA. They concluded that with beta-adrenergic stimulation, phosphorylation of cMyBP-C is mainly responsible for the effect to increase LDA and the Frank-Starling mechanism.

## 4. Modulation of sarcomere function by S-glutathionylation of cMyBP-C, actin, cTnI, and titin

### 4.1. Functional effects of S-glutathionylation of cMyBP-C, actin, and cTnI

To our knowledge, our determination of effects of oxidative stress in a DOCA-salt mouse model with unilateral nephrotomy provided the first report in 2012 of a functional effect of cMYBP-C S-glutathionylation (4). The DOCA-salt model exhibits slight hypertension with diastolic dysfunction and preserved ejection fraction. Cardiac myocytes isolated from the DOCA-salt model had no differences from controls in Ca^2+^-currents, Ca^2+^-transient dynamics, and late Na^+^ currents. These results indicated an altered myofilament Ca^2+^-response. We therefore compared properties of detergent-extracted (skinned) fiber bundles from controls and DOCA-salt hearts. We measured tension and ATPase activity simultaneously over a range of Ca^2+^ concentrations. Compared with controls, fiber bundles from DOCA-salt hearts showed an increase in Ca^2+^-sensitivity and a decrease in the slope of plots of tension vs. ATPase rate indicating a reduction in tension cost (unit tension developed/unit ATP hydrolyzed) and slowing of cross-bridge kinetics. Determination of Ktr (the transition of cross-bridges into the force generating state) showed no differences between controls and DOCA-fibers. These findings supported our conclusion that the diastolic dysfunction in the DOCA-salt hearts was due to a slowing of cross-bridge in their exit from the force generating state. We hypothesized that the oxidative stress induced post-translational modification in the myofilament proteins. We found no differences in phosphorylation among the proteins in controls and DOCA-fibers, but there was an S-glutathionylation of cMyBP-C that correlated with the extent of change in tension cost. In subsequent experiments we confirmed these results in which we treated the DOCA-salt mice with tetra-hydro biopterin (BH4) to suppress the oxidative stress (58). Inasmuch as oxidative stress affects multiple pathways, we tested for direct effects of S-glutathionylation of myofilament tension and ATPase activity by incubating skinned fibers and myofibrillar fractions with oxidized glutathione (GSSG) (59). We also determined sites of S-glutathionylation employing mass spectrometry. As with the preparations isolated from DOCA-salt hearts, myofilaments incubated in GSSG had an increased Ca^2+^-sensitivity. Sites of S-glutathionylation as determined by mass spectrometry occurred at cysteines 655, 479, and 627.

Follow-up studies comparing samples of donor controls with samples of human hearts with dilated and ischemic cardiomyopathy extended and confirmed these results and identified a potential interaction between S-glutathionylation and protein phosphorylation that appeared mutually exclusive. In 2016, Stathopoulou et al. (43) reported a correlation with a decrease in site specific phosphorylation of cMyBP-C with increases in S-glutathionylation of cMyBP-C. In general agreement with this study, Budde et al. (44) reported studies of a role S-glutathionylation of myofilaments in single cardiac myocytes harvested from human heart samples from control donors and patients with end-stage heart failure (HF). As reported previously they found the HF cells had reduced maximum tension and Ca-sensitivity. By treating the myocytes with reduced GSH they were able to correct these changes. They found increased S-glutathionylation of both cTnI and cMyBP-C in the HF samples compared to the donor samples. Treatment with PKA failed to phosphorylate these proteins in the HF samples but worked in the donor samples. Moreover, there was increased titin stiffness in the HF samples that could be suppressed by treatment with PKA and GSH. These data indicated a significant role for interactions of S-glutathionylation and phosphorylation in end stage human heart failure.

In another study extending understanding of a role of S-glutathionylation in cardiac function, Cazorla and colleagues compared redox related modifications in sedentary rats and rats stressed with moderate and exhaustive, high stress exercise (HSE) (45). Compared to controls, bouts of HSE induced large changes in oxidative stress markers together with depressed diastolic and systolic function in isolated perfused hearts. Hearts of HSE mice also demonstrated significantly increased S-glutathionylation of cMyBP-C and a depression cMyBP-C phosphorylation that could not be increased by activation of PKA. There was also a decrease in cTnI phosphorylation although there was no report of S-glutathionylation of cTnI. These data fit with findings in human hearts samples at end stage failure (43).

There are several lines of evidence indicating that post-translational modification of actin by S-glutathionylation may also be an important factor in control of cardiac dynamics. However, effects of actin S-glutathionylation on sarcomere function have not been a consistent finding. An early report by Chen and Ogut (60) identified S-glutathionylation of cardiac actin associated with ischemia suggesting this effect as a possible contributor to the decline in function resulting from myocardial infarction (60). Follow up studies reported that compared to controls there was a depression of acto-myosin S-1-ATPase activity of *in vitro* reconstituted preparations containing S-glutathionylated actin (61). In support of these findings, Passarelli et al. (3) reported a depression in force generation of cardiac myofibrils treated with agents promoting S-glutathionylation. Analysis of mechanism indicated actin S-glutathionylation as the main effector of this depression. Human heart samples analyzed by histochemical techniques showed evidence for *in situ* actin S-glutathionylation. A depression in actin polymerization has also been reported as an associated effect of actin S-glutathionylation at Cys 374 (60, 62). In our investigation of sites of S-glutathionylation determined by mass spectrometry together with functional effects on tension and ATPase activity of cardiac skinned fibers and myofibrils incubated with GSSG, we found no evidence of S-glutathionylation of cardiac actin at Cys 376 (59). There was a correlation of cMyBP-C S-glutathionylation with increased Ca-response of tension and ATPase activity with no change in maximum or minimum values. Moreover, we found no evidence of actin S-glutathionylation in hearts expressing the HCM linked mutant TmE180G discussed below. A main conclusion from publications from our and other laboratories is a dominant and consistent modification of sarcomere proteins by S-glutathionylation is at cMyBP-C and titin. Existing evidence does indicate a need for further investigation of the potential role of actin-S-glutathionylation of integrated function of cardiac myocytes.

### 4.2. Functional effects of S-glutathionylation of titin

Investigations of possible role of S-glutathionylation in the control of stiffness of cardiac titin began with oxidative stress by acute coronary artery occlusion in a mouse acute myocardial infarction model (63). Taking note of this finding, Alegre-Cebollada et al. (64) established a collaboration with our laboratory in confirming and extending this finding. The studies employed a fragment of I-band titin region rich in Cys residues as illustrated in Figure 3. Stretch of the fragment exposed cryptic Cys residues making them substrates for GSSG and inducing S-glutathionylation. This modification decreased the mechanical stability of the domain and suppressed folding ability. Studies with isolated human cardiac myocytes demonstrated that this effect is translated to the intact system thereby enhancing titin elasticity (64). These results provide new understanding of the effects of oxidative stress on titin compliance, which had been reported to be due largely to S-S bonds between clusters of Cys residues. In the case of Cys residues of Ig domains the Cys residues are separated enough to be substrates for GSSG when exposed by stretch. Loescher et al. (12) investigated multiple models of oxidative stress together with altered mechanical unloading in an extensive investigation in which they described this mechanism as unfolded domain oxidation (UnDOx). Important findings described in detail in the publication provided evidence that with altered mechanical loading of cardiac myocytes, UnDOx in a distal titin spring region can either up or down regulate titin compliance affecting passive tension and enhancing titin phosphorylation. These findings established the relative significance of UnDOx in regulation of passive tension and thereby filling of the ventricle with blood in diastole. Modifications in titin phosphorylation have been reported with oxidant stress, but whether there is an interaction between S-glutathionylation and phosphorylation remains unclear (65).

## 5. Avenues of future research in cardiac physiology and pathology

### 5.1. Sarcomere protein S-glutathionylation in hypertrophic cardiomyopathy (HCM)

Results of our investigations of oxidative stress induced S-glutathionylation of cMyBP-C in a mouse model of hypertrophic cardiomyopathy expressing αTm-E180G (Tm-180) provided insights into the complexities of this PTM in a common cardiac disorder. Compared to wild-type, myofilaments controlled by Tm-180 have increased Ca-sensitivity. This biophysical signal triggers a pathological progression inducing diastolic dysfunction with preserved ejection fraction, oxidative stress, hypertrophic signaling with remodeling, and fibrosis. There is also oxidative stress together with S-glutathionylation of cMyBP-C because of increased eNos activity. We approached suppressing the oxidative stress either by treatment with NAC (N-acetyl cysteine; promotes GSH synthesis) (66, 67) or with FTY-720 (Fingolimod) (68). FTY-720 is an immuno-modulator approved for use in multiple sclerosis. We previously reported FTY-720 to be effective reducing remodeling and improving function in a pressure-overload mouse model (69). FTY-720 affects sphingolipid signaling by interaction with receptors for sphingosine-1-phosphate. FTY-720 treatment reduced the elevated serum leucocyte population occurring in Tm-180 mice. In both approaches there was an improvement in diastolic dysfunction and a reduction in oxidative stress that was correlated with a reduction in levels of cMyBP-C S-glutathionylation. With FTY-720 treatment there was no change in phosphorylation of sarcomere proteins including total cMyBPC, titin, and cTnI. Moreover, restoration of diastolic function to control levels occurred with no change in fibrosis. However, in the study with NAC treatment, site specific phosphorylation of cTnI at Ser 23 and Ser 24 was increased in the controls but not in the Tm-180 mice. We found no S-glutathionylation of cTnI suggesting that in this case altered kinase or phosphatase activity was in play and not direct interference of phosphorylation by S-glutathionylation. We also found a significant increase in site specific phosphorylation of cMyBP-C at Ser 282 that occurred in the Tm-180 filaments and was reduced substantially with NAC. These results indicate that levels of sarcomere protein phosphorylation may be directly affected by alterations of Cys residues by S-glutathionylation, but the net effects may also depend on oxidative stress effects on kinase/phosphatase pathways (70).

Apart from the suggestions above about use of serum levels of S-glutathionylated cMyBP-C in diagnosis and patient stratification, there are several gaps in our understanding of the role of S-glutathionylation of major sarcomere proteins in cardiac physiology and pathology. The unique ability of S-glutathionylation to modify Cys residues reversibly and to protect against irreversible oxidative modifications needs to be more completely understood in relation to physiological processes for example, in exercise and aging. Whereas correlations with diastolic dysfunction remain a strong possibility, there is evidence that this PTM may occur in a variety of cardiac disorders many of which display oxidative stress. More studies on early-stage cardiac diseases are in order. These studies need to integrate S-glutathionylation in the major affected proteins, cMyBP-C and titin as well as cTnI.

We think an important unexplored avenue of research is S-glutathionylation induced modulation of sarcomere length dependent activation (LDA), i.e., the Frank-Starling mechanism. Evidence of a role for titin (10, 71), cMyBP-C (56, 57), and cTnI (41, 55, 57) in altering LDA provides a rationale for this suggestion. A further rationale that is unexplored is the possible modification of LDA in the oxidative stress associated with cardiac disorders demonstrating mechanical dyssynchrony. Early activation of the septum associated ith late activation of the left ventricular free wall characterizes dyssynchronous heart failure (DHF). A common cause is abnormal Purkinje fiber conduction. However, there is also evidence that DHF occurs with altered sarcomere mechano-biochemical signaling. Although bi-ventricular pacing can suppress the DHF, a significant number of patients do not respond to this treatment. Understanding of this lack of responsiveness may involve abnormal LDA and engagement of the Frank-Starling mechanism.

In testing patients with non-obstructive but symptomatic HCM, Ahmed et al. (72) applied CRT (bi-ventricular pacing, BiV) and tested for improvements in LV diastolic filling and exercise capacity. Their approach was based on evidence of a significant incidence of dyssynchrony in HCM patients without obstructive outflow disorders. The dyssynchrony manifests at rest in a maladaptive interaction between the RV and LV in diastole inducing a failure of the LV to fill properly during exercise resulting in a failure to employ the Frank-Starling mechanism. After 8 months of sham or BiV, there was an improvement in LV volume during exercise, an increase in exercise capacity, and quality of life scores. The authors did not investigate molecular mechanisms but did evaluate the effects of BiV on LV contractility. At rest, there was no change in mechanical dyssynchrony in the study groups and no change in systolic elastance associated with BiV indicating no change in contractility. Thus, with some allusions to limitation of the study, the authors conclude that major mechanisms for the improvements in exercise were the effect of BiV to engage the Frank-Starling mechanism. Inasmuch as length dependent sarcomere activation is the basis of the Frank-Starling mechanism, this conclusion emphasizes consideration of LDA in sarcomeres with HCM-linked mutations.

The significance of LDA in CRT has been investigated by Niederer et al. (73). By employing a patient specific model of excitation and mechanics from the level of cellular mechanisms to heart function determined in patients before and after CRT. Sensitivity of the model to various elements in cellular control mechanisms demonstrated LDA as a significant contributor to the therapeutic benefit of CRT. The computations indicated a reduced effect of CRT in patients with dysfunctions in LDA. A significant contribution of these findings is the idea that the Frank-Starling relation is not only important in beat-to-beat control of cardiac function but a significant mechanism in synchrony of the heartbeat. Mechanical LDA and other sub-cellular mechanisms are downstream to electrical stimulation and thus dampened or amplified LDA appears to be a relatively important contributor to the efficacy of CRT. This study indicates that despite the electrical effects of CRT, it is critical for the heart to have robust LDA.

Our studies with models of HCM are relevant to these concepts. Early studies from our lab investigated the LDA of myofilaments expressing cTnT-R92Q. The data indicated a higher level of LDA in the R92Q skinned fibers compared to WT fibers (74). Other studies reported an attenuation of LDA in HCM models cTnT-R95H and cTnT-F88L (75, 76). It would be of interest to determine a role of oxidative stress and potential S-glutathionylation of sarcomere proteins in these models.

### 5.2. Potential use of S-glutathionylated sarcomere proteins as biomarkers of oxidative stress and diastolic dysfunction

Reports of determination of cMyBP-C and its fragments as a biomarker for cardiac disorders provide a proof of concept that determination of serum levels of S-glutathionylated cMyBP-C may be a biomarker with functional implications. Studies identified serum cMyBP-C as a biomarker of severe cardiovascular diseases and in acute myocardial infarction (77–80). Based on our studies of S-glutathionylation of cMyBP-C in a model of diastolic dysfunction, Dudley and colleagues (81) further investigated changes in cMyBP-C in cardiac samples of mice fed on normal chow or a high fat diet (HFD) inducing diastolic dysfunction and insulin resistance. One group of mice were also given Mito TEMPO, an investigational agent not approved by the FDA, concurrently to suppress mitochondrial induced oxidative stress. The results showed an increase in S-glutathionylation of cMyBP-C with the HFD that was reversed or prevented in the mice on the HFD with mito TEMPO. These data encouraged an effort to develop antibody detection of S-glutathionylated cMyBP-C using an immune-precipitation approach in serum of models of cardiometabolic syndrome and in humans (82). Compared to controls with no diastolic dysfunction, levels of serum S-glutathionylated cMyBP-C were elevated in the serum of mice, monkeys, and in a cohort of 24 humans with diastolic dysfunction. These promising preliminary data require verification in larger cohort of patients and measurements need to be made to determine specificity, and biological variation in healthy individuals as has been carried out for comparisons of cTnT and cMyBP-C (80). Knowledge of epitopes employed in antibody detection as well as quantification of levels of S-glutathionylation in relation to severity of disease. The vast literature on serum cTnI and cTnT as biomarkers (83) provides an example of the dimensions of the information and effort required to establish confidence in the use of S-glutathionylated cMyBP-C as a serum biomarker for cardiac disorders. An issue requiring further investigation is the stability of S-glutathionylated cMyBP-C in muscle and serum samples. To reduce thiol oxidative degradation in serum samples, non-reducing preservative reagents, N-Methylmaleimide, neocurporine, and diethylenetriaminepentaacetic acid were added to samples in the determination of serum levels of cMyBPC- prior to binding to primary antibodies (82). We have investigated the stability of post-translational modifications of cardiac/myofibril samples from hearts of mice treated with different euthanizing agents. Our findings indicate a fall in levels of glutathionylation of cMyBP-C between 30 and 90 days of storage (84).

## 6. Summary and conclusion

Results of studies presented here show progress identifying S-glutathionylation of sarcomeric proteins in advanced understanding of physiological stressors and common cardiac disorders. An example is insights into the relation between mechano-biology and S-glutathionylation of titin that provide a novel perspective on relations between protein folding and oxidative stress. This finding may have application to mechano-biology and S-glutathionylation of many other proteins including cMyBP-C, which expresses stretch sensitive regions analogous to the PEVK region of titin (85). Studies summarized here also provide an account of translation of laboratory experiments on S-glutathionylation of sarcomere proteins to clinical applications. Among the various sarcomere modifications discussed, the data indicate that S-glutathionylation of cMyBP-C is a significant regulator of cardiac function and the most promising candidate to add to the list of biomarkers now in use or development. We have stressed S-glutathionylated cMyBP-C as a biomarker in metabolic syndrome and HFpEF, but other uses are evident in cardiac disorders with oxidative stress as a hallmark of the pathology. As we have discussed previously in the case of use of troponins as cardiac injury biomarkers, it is possible that the S-glutathionylated cMyBP-C biomarker may find use in conditions such as cardio-renal syndrome or in SARS-CoV-2 infections (83, 86). We think our discussions here provide a rationale for continued investigation of this powerful post-translational mechanism in sarcomere proteins.

## Data availability statement

The original contributions presented in this study are included in the article/supplementary material, further inquiries can be directed to the corresponding author.

## Author contributions

RS decided on the topic idea and after discussions with PR wrote a first draft. PR read and edited the first draft and finalized the manuscript with the RS. Both authors contributed to the manuscipt and approved the submitted version.
